# Auxin as a player in the biocontrol of Fusarium head blight disease of barley and its potential as a disease control agent

**DOI:** 10.1186/1471-2229-12-224

**Published:** 2012-11-22

**Authors:** Carloalberto Petti, Kathrin Reiber, Shahin S Ali, Margaret Berney, Fiona M Doohan

**Affiliations:** 1 Molecular Plant-Pathogen Interaction Group, School of Biology and Environmental Science, University College Dublin, Science Education and Research Centre West, Belfield, Dublin, Ireland; 2 Current address: Department of Horticulture, Science Centre North, University of Kentucky, Lexington, Kentucky, 40502, USA; 3 UCD School of Biology and Environmental Sciences, Room 148, Science Education and Research Centre West, UCD, Belfield, Dublin 4, Ireland

**Keywords:** Hormone, IAA, ABA, *Pseudomonas fluorescens*, Biocontrol, Fusarium head blight

## Abstract

**Background:**

Mechanisms involved in the biological control of plant diseases are varied and complex. Hormones, including the auxin indole acetic acid (IAA) and abscisic acid (ABA), are essential regulators of a multitude of biological functions, including plant responses to biotic and abiotic stressors. This study set out to determine what hormones might play a role in *Pseudomonas fluorescens –*mediated control of Fusarium head blight (FHB) disease of barley and to determine if biocontrol-associated hormones directly affect disease development.

**Results:**

A previous study distinguished bacterium-responsive genes from bacterium-primed genes, distinguished by the fact that the latter are only up-regulated when both *P. fluorescens* and the pathogen *Fusarium culmorum* are present. *In silico* analysis of the promoter sequences available for a subset of the bacterium-primed genes identified several hormones, including IAA and ABA as potential regulators of transcription. Treatment with the bacterium or pathogen resulted in increased IAA and ABA levels in head tissue; both microbes had additive effects on the accumulation of IAA but not of ABA. The microbe-induced accumulation of ABA preceded that of IAA. Gene expression analysis showed that both hormones up-regulated the accumulation of bacterium-primed genes. But IAA, more than ABA up-regulated the transcription of the ABA biosynthesis gene *NCED* or the signalling gene *Pi2,* both of which were previously shown to be bacterium-responsive rather than primed. Application of IAA, but not of ABA reduced both disease severity and yield loss caused by *F. culmorum,* but neither hormone affect *in vitro* fungal growth*.*

**Conclusions:**

Both IAA and ABA are involved in the *P. fluorescens-*mediated control of FHB disease of barley. Gene expression studies also support the hypothesis that IAA plays a role in the primed response to *F. culmorum.* This hypothesis was validated by the fact that pre-application of IAA reduced both symptoms and yield loss asssociated with the disease. This is the first evidence that IAA plays a role in the control of FHB disease and in the bacterial priming of host defences.

## Background

Biological control bacteria regulate the capacity of plants to resist pathogen attack through diverse mechanisms [1-6]. Recently, Zhang *et al.* (2011) showed that bacteria-responsive plant microRNAs regulate plant innate immunity by modulating the plant hormone network [7]. Phytohormones are key determinants of a plants’ ability to tolerate abiotic and biotic stress (reviewed by [8]). They are the effector molecules responsible for signal perception/transduction, cellular homeostasis and gene expression. As a consequence, they play an important role in plant responses to, and resistance against, disease.

Hormones and hormone-synthetic analogs have been used to prime plants such that they are prepared to mount defence responses against various pathogens. The induced systemic resistance (ISR) pathway is stimulated during necrotrophic bacterial attack and is primed by biocontrol species such as *Pseudomonas* to protect the plant from pending attack, possibly by increasing the plants susceptibility to the ISR-regulating hormone jasmonic acid (JA) [9]. The priming of the ISR response is linked to JA and ethylene (ET), yet significant accumulation of these hormones has not been reported in ISR-expressing plants [3]. Bacterium-mediated ISR is not dependent upon the induction of the defence hormone salicylic acid (SA), at least for the rhizobacterium strain *P. fluorescens* WCS417r [10,11]. Application of the auxin β-aminobutyric acid resulted in induced resistance to *Alternaria brassicola* and priming for callose deposition and resistance [12]. Priming of callose deposition was dependent on the classical plant defence hormone abscisic acid (ABA). Other hormones such as the the auxin indole acetic acid (IAA), cytokinins and brassinosteroids modulate host defence responses [13], but have not yet been specifically linked to defence priming.

A number of *P. fluorescens* strains have already been reported to prime plants by initiating defence responses to subsequent pathogen attacks [4]. *P. fluorescens* strain MKB158 had the ability to induce local and systemic responses in wheat and barley tissue, resulting in enhanced resistance to Fusarium seedling blight and head blight (FSB and FHB) disease [14-17]. The objective of this study was to determine what hormones are involved in the defence responses to FHB in barley that are primed by the bacterium *P. fluorescens* strain MKB158. Based on *in silico* analysis of the upstream regions of genes involved in the primed response [17] we chose to determine whether the hormones ABA and IAA play a role in the biocontrol of FHB disease by *P. fluorescens*. Based on the assessment of both hormone levels and their effect on both the regulation of plant genes activated by the biocontrol bacterium and the development of FHB disease symptoms, we draw conclusions regarding the contribution of IAA and ABA to the local defence responses of barley plants primed by *P. fluorescens*. Furthermore, we highlight the potential of IAA as a treatment for the control of FHB disease.

## Results

### ABA- and GA-responsive elements are highly represented in potentiated genes

We previously identified 86 barley genes that were primed by *P. fluorescens* to respond to *F. culmorum*[17]. Barley genome sequence was available for 39 (45%) of these genes (see Additional file 1: Table S1). *In silico* analysis of upstream promoter regions indicated that 38 of these contained hormone-responsive elements (Additional file 1: Table S1). ABA- and GA-responsive elements were detected in all of these upstream regions, ABA being most commonly detected (between 1–12 ABA-responsive elements per upstream region analysed) (Additional file 1: Table S1). Elements responsive to the hormones AUX/IAA and SA were also frequent, being detected in 64 and 56% of putative promoter regions, respectively. JA-responsive elements were identified within the upstream region of a histone *H4* gene and genes involved in defence - glutathiones, peroxidases, *MLA*12 and PDR-type ABC transporters.

### ABA and IAA levels are modulated by both the biocontrol agent and the pathogen

**Figure 1:**
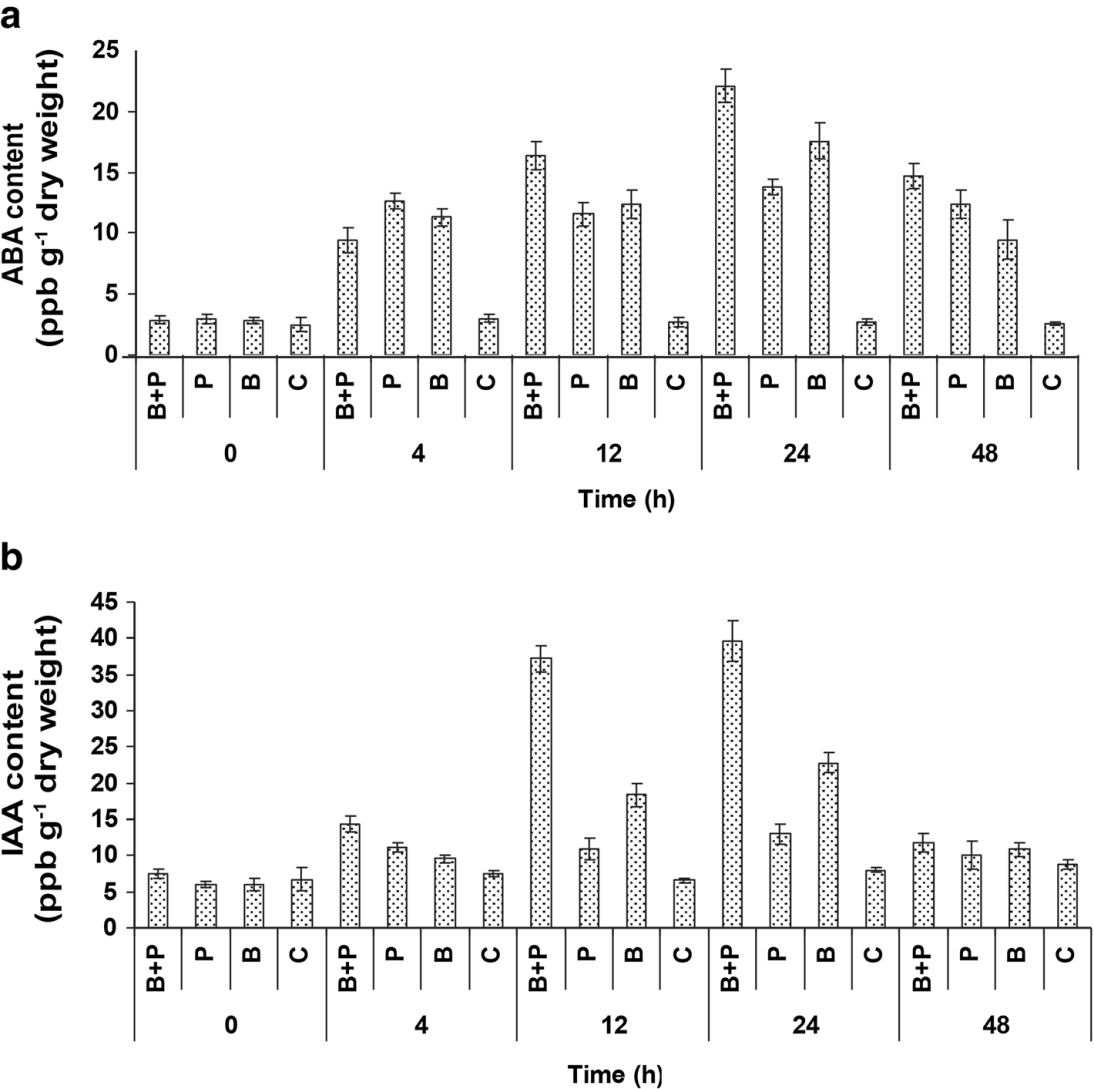
**Effect of pre-treatment with *Pseudomonas fluorescens *strain MKB158 on the accumulation of (a) abscisic acid (ABA) and (b) indole-3-acetic acid (IAA) in barley head tissues inoculated with *Fusarium culmorum. *** Heads were treated with cells of *P. fluorescens* strain MKB158 or water 24 h pre-inoculation with conidia of *F. culmorum* strain FCF 200. Heads were harvested at various time points post-pathogen treatment, hormones were extracted and quantified by ELISA analysis. Treatments codes: C, controls treated with water and Tween20; B, bacterium plus Tween20; P, pathogen plus Tween20; B+P, bacterium plus pathogen. Results are based on two biological replicates, each containing 4 bulked technical replicates per treatment. Bars indicate standard deviation.

Experiments were conducted in order to determine if ABA or IAA accumulation varied during the early stages of FHB disease development in barley and if this was affected by application of the biocontrol bacterium *P. fluorescens* (24 h pre-pathogen treatment)*.* ABA was produced in response to both bacterium and fungal treatment as early as 4 h post-pathogen treatment. ABA production (Figure 1a) was induced by both bacterial and fungal treatment. Noteworthy was the fact that at any of the time points tested, ABA levels in plants treated with both the bacterium plus fungus were not significantly different from the levels determined in the bacterium-treated plants (*P*>0.05). The combined effects of both agents on ABA accumulation were neither additive nor synergistic. ABA production peaked at 24 h post-fungal inoculation and by 48 h it had not returned to basal levels.

Similar to ABA, IAA was produced in response to both *P. fluorescens* and *F. culmorum* as early as 4 h post-pathogen treatment. Unlike ABA, additive effects of the bacterium and fungus on hormone accumulation could account for the levels of IAA detected in plants treated with both agents at 24 h; at 12 h effects were at least additive and potentially synergistic (Figure 1b). By 12 h post-*Fusarium* inoculation, IAA production levels were 2.8-fold higher in bacterium as compared to control samples and 1.3-fold greater than the fungus samples (*P*<0.05). But IAA levels were 5.8-fold higher in plants treated with both bacterium and fungus as compared to control plants (*P*<0.05). Similar results were observed at 24 h post-fungal inoculation. But, by 48 h post-fungal inoculation, IAA levels were similar across all treatments (P>0.05) (Figure 1b).

### Bacterium-potentiated genes are up-regulated in response to both IAA and ABA

**Figure 2:**
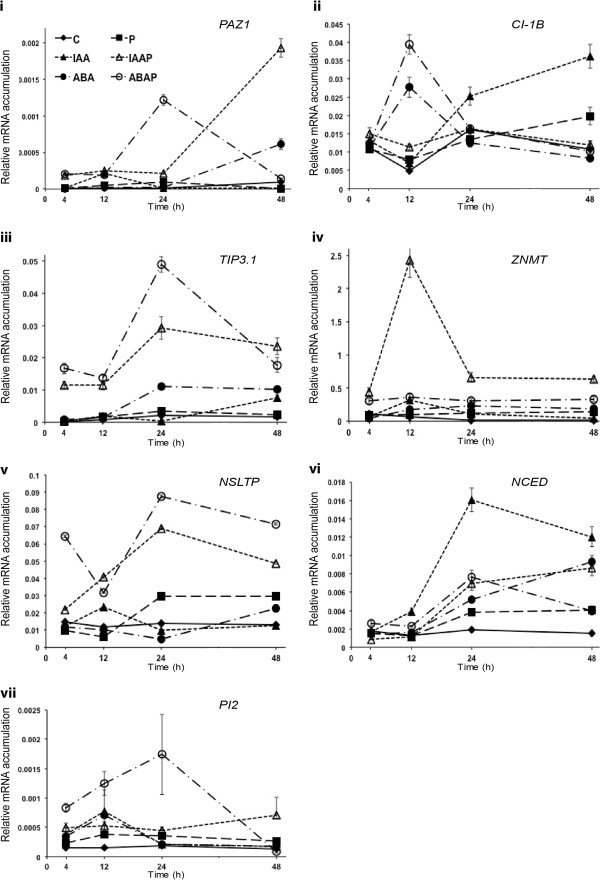
**Temporal analysis of the effect of hormones abscisic acid (ABA) and indole-3-acetic acid (IAA) with or without application of pathogen *Fusarium culmorum *on the accumulation of select transcripts in heads of barley cultivar Lux.** Transcripts represented are: (**a**) serpin Z4 (*Paz1*), (**b**) subtilisin-chymotrypsin inhibitor (*CI-1B*), (**c**) tonoplast aquaporin (*TIP3:1*), (**d**) zinc methallothionin-like protein (*ZnMT*), (**e**) putative non-specific lipid transfer protein (*nsLTP*), (**f**) nine-cis-epoxycarotenoid dioxygenase *NCED* and (**g**) a signalling cascade protein (*Pi2*). Transcripts were previously identified by microarray analysis as being primed by the bacterium to accumulate in response to the pathogen at either 24 or 48 h post-pathogen treatment (**a** to **e**) or as being activated by the bacterium only (**f** and **g**). Treatments: barley heads were treated with IAA, ABA or Tween20 (control treatment), and 24 h later, with pathogen (P) or Tween20. RNA extracted from treated head tissue at either 4, 12, 24 or 48 h post-hormone or hormone and pathogen treatment was used for real-time RT-PCR analysis. ^a^Transcript accumulation was quantified as 2^-(CT target transcript–CTα-tubulin)^. Treatment codes: C, controls treated with Tween20; P, pathogen (*F. culmorum*); IAA, indole-3-acetic acid; ABA, abscisic acid; IAA + P; IAA plus pathogen; ABA+P, ABA plus pathogen. Results are based on two biological replicates, each including two techical replicates per treatment. Bars indicate standard error of mean.

Previously we discriminated two set of plant genes activated in response to *P. fluorescens* strain MKB158; one set were transcriptionally activated by the bacterium alone and the other set were primed by the bacterium to respond to the pathogen *F. culmorum,* respectively known as bacterium-responsive and bacterium-potentiated genes [17]. We hypothesised that if either ABA or IAA were involved in priming then, in pathogen-treated heads, they may up-regulate potentiated genes but not necessarily bacterium-responsive genes. Potentiated genes studied were defence genes *nsLTP*, *CI-1B*, *Tip3:1*, Paz1 and *ZnMT*[17]. Bacterium-responsive genes studied were *NCED* (a fundamental gene involved in the initial steps of ABA biosynthesis; [18,19] and a protein kinase *Pi2*[20]. We assessed the effect of both IAA and ABA on the expression of the aforementioned potentiated and bacterium-responsive genes in control (Tween20) and *F. culmorum* treated barley heads from 4 to 48 h post-treatment (Figure 2). Gene expression was highest at 12 – 24 h post-treatment with pathogen or Tween20 (controls). In the absence of the pathogen, both ABA and IAA up-regulated most genes but transcript levels were generally much lower than in pathogen-treated heads (Figure 2). In pathogen-inoculated heads, both IAA and ABA significantly up-regulated the potentiated genes and *NCED* (*P*<0.05) (Figure 2a – 2f). *PI2* was potentiated by ABA and by IAA to respond to *F. culmorum*, although the effects of ABA were more immediate and greater (Figure 2g). ABA effects on gene expression in pathogen-treated tissue were generally more immediate than those of IAA, with the exception of *ZnMT* and *NCED*. *ZnMT* was the most IAA-responsive gene; it was the transcript that accumulated to the highest levels and, by 12 h post-fungal treatment, it was up-regulated 24 fold in heads treated with *Fusarium* plus IAA as compared to *Fusarium* alone (*P*<0.05) (Figure 2d).

### Exogenous application of IAA reduces FHB disease in barley

**Figure 3:**
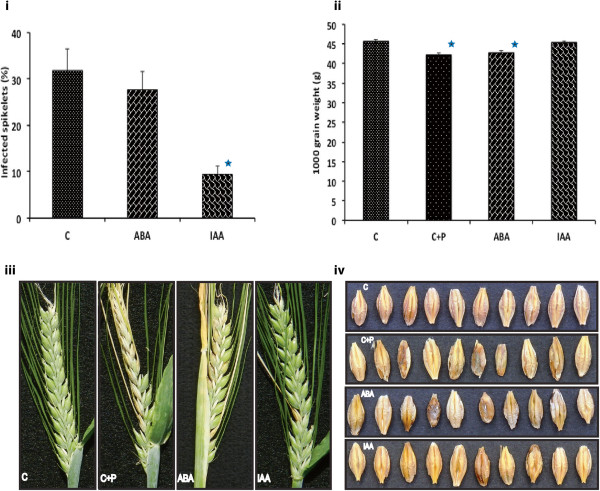
**Exogenous application of IAA and ABA on FHB progression on barley treated heads and its effects on seed grain yields and quality.** (**a**) Percentage of infected spikelets following IAA and ABA pre-Fusarium treatment. (**b**) A 1000 grain weight determined for the untretad water control (C) and the corrisponding pathogen exposed-IAA and ABA and the no-treatment head (C+P). (**c**) Fusarium head blight effects on barley heads for untreated control (C+P), IAA, ABA as compared to unexposed control (C). (**d**) Fusarium head blight effects on barley kernels. Treatment codes: C, controls treated with Tween20; C+P, controls treated with Tween20 plus pathogen (*F. culmorum*); IAA, indole-3-acetic acid; ABA, abscisic acid. Bars indicate standard error of mean.

Experiments were conducted in order to determine the effect of IAA and ABA application to barley heads on both visual symptoms and yield loss caused by the subsequent inoculation with *F. culmorum.* Pathogen inoculation resulted in 30% of spikelets displaying symptoms by GS 80 and a 7.6% reduction in 1000 grain weight (*P<*0.001, Figure 3). ABA did not significantly reduce the disease symptoms or yield loss caused by the pathogen (*P*>0.05, Figure 3). On the contrary, IAA reduced disease levels by 60% and negated the yield losses caused by the pathogen (*P*<0.001). Grains were visually similar to those obtained for control plants (non-pathogen treated); in general they did not display the fungal growth and shrinkage evident in grains treated with pathogen (positive control) or ABA + pathogen (Figure 3d).

### No evidence that IAA and ABA directly inhibit fungal growth *in vitro*

An *in vitro* plate assay was conducted in order to assess the effect of hormone supplementation on PDA on the growth of *F. culmorum* strain FCF200 (at 72 h post-inoculation). Irrespective of the hormone concetration used, neither IAA nor ABA significantly affected the growth of the fungus, relative to control plates (Additional file 2: Figure S1).

## Discussion

The importance of the hormones IAA and ABA as effectors molecules involved in the regulation of a variety of plant physiological mechanisms is well reported (reviewed in [8]. Moreover, an increasing body of evidence implicates a dualistic role of these hormones in plant defences and sensitivity to pathogens [21-26]. However, very little is known regarding their modulation by biocontrol agents. Herein we show that a biocontrol pseudomonad modulates IAA and ABA levels, that IAA and ABA either directly or indirectly influence the transcriptional regulation of genes involved in the response of the barley plants to these microbes, and that IAA can reduce the severity of FHB disease. The promoters of many of the bacterium-potentiated genes also possessed both SA and JA-responsive elements in addition to those responsive to ABA and IAA. While JA plays a key role in ISR, SA is classically a SAR-associated hormone [27]. The SA response is generally concomitant with down-regulation of IAA [28,29] the most abundant SA-responsive element within the promoters of bacterium-potentiated genes was TGACG, and this element is also responsive to IAA, biotic and abiotic stimuli [30,31]. This motif is present in the *A. thaliana* NPR1 gene promoter [32], the product of which regulates both ISR (SA-independent) and SAR responses [33].

Auxin production was part of the response primed by *P. fluorescens* to respond to *Fusarium* infection and IAA reduced the severity of disease symptoms and associated yield loss when applied 24 h pre-pathogen. The increases in IAA observed herein in biocontrol plus pathogen treated tissue precede disease spread and thus are part of an early priming cascade activated in response to plant-pathogen contact. We found no evidence that either IAA or ABA inhibited fungal growth on PDA. *P. fluorescens* strain MKB158 has the capacity to produce auxin [34], as do *Fusarium* fungi [35]. Based on the literature [36-38], it is very possible that the IAA is derived from the bacterium and/or pathogen rather than the plant; the application of IAA might trick the plant into thinking it is being attacked by the pathogen, thus priming the defences responses such that they are activated to respond rapidly to the pathogen and thus increase plant FHB resistance. *Fusarium culmorum* displays a hemibiotrophic lifestyle [39] and there is evidence that auxin signalling enhances resistance to necrotrophs but susceptibility to biotrophs [40-42]. Noticeably, a recent report [43] highlights how auxin application was able to reverse hypersensitive response programmed cell death in tobacco initiated by a *Erwinia amylovora* type III elicitor harpin [43]. In a recent metabolomics study reports by Kumaraswamy et al. (2012), *F. graminearum* induced the accumulation of IAA in barley [44].

Most evidence points to ABA playing a negative role in plant disease resistance, although there are exceptions, particularly for necrotrophic pathogens (reviewed by Cao et al. [45]). But, it has a negative effect on some necrotrophs and therefore the role of ABA in the host response in not solely determined by the pathogen lifestyle. The predominance of ABA-responsive elements in the upstream region of primed genes, the previous association between ABA and primed biocontrol responses [46], its association with diverse pathogen resistances [47] and the increased ABA levels in bacterium and pathogen treated plants all provide “circumstantial” evidence that this hormone might play a role in the interplay between *Fusarium* and barley. Furthermore, there is evidence for a link between ABA and FHB resistance: callose deposition and repression of ethylene signalling are associated with ABA [12] and also with resistance to *Fusarium* spread in wheat spikelets [48-50]. Both the biocontrol agent and pathogen induced the accumulation of similar levels of ABA, indicating that any quantitative effects of this hormone on biotic responses are general rather than organism-specific. ABA itself did not reduce the severity of FHB. IAA activates the expression of the ABA biosynthetic gene *NCED,* as found by us and others [51]. Thus it is conceivable that application of IAA activates ABA-associated defences, but the converse does not occur; this warrants investigation. It could also be that the timing of application of ABA and the activation associated defence cascades were not optimal for disease control.

## Conclusions

Defence responses are finely modulated by multiple hormones and herein we report of how the application of a biocontrol bacteria activated the *in planta* biosynthesis of two fundamental hormones, ABA and IAA. Studies that focus on the effect of specific components of IAA biosynthesis, homeostasis and turnover on defence priming will greatly enhance our understanding of how biocontrol pseudomonads can be used more effectively to control plant diseases. This study has identified IAA as a method for controlling FHB disease. Other studies have shown that IAA can reduce the severity of Fusarium seedling blight disease of wheat and barley (Khan et al., unpubl. data). Interestingly, it has been associated with systemic acquired immunity [52] and it has protective effects against apple scab caused by the necrotroph *Botrytis cineria* when applied pre- but not when applied post-pathogen [53]. IAA might offer a realistic treatment for the control of diseases such as FHB where crops have a limited and clearly defined infection (mid-anthesis for FHB). Thus the effect of IAA on mycotoxin accumulation in grain and on other agronomic parameters are worthy of investigation.

## Methods

### Maintenance and culture of microorganisms

The biocontrol bacterium used in this study was *Pseudomonas fluorescens* strain MKB158; this bacterium was chosen because of its ability to control FSB and FHB diseases of wheat and barley and to reduce mycotoxin contamination in the grain [14,15]. Culture conditions and inoculum preparation were as described in [14]. The phytopathogenic *Fusarium culmorum* (W. G. Smith) Sacc. strain FCF 200 (kindly supplied by Dr. Paul Nicholson, John Innes Center, Norwich, UK) was grown at 24°C on PDA plates. The maintenance of *F. culmorum* and the production of conidial inoculum (10^5^ conidia ml^-1^ 0.2% Tween20) were as described earlier [14].

### *In silico* analysis of gene promoters

We previously identified genes that were primed by *P. fluorescens* to respond to attack by *F. culmorum*[17]. These were discriminated as transcripts differentially regulated only when both agents, *i.e*. the biocontrol bacterium and the pathogen, are present and are herein and after referred throughout the manuscript to as potentiated (see Additional file 1: Table S1 in [17]). The probe sequence corresponding to the potentiated genes was retrieved from the plant expression database (http://www.Plexdb.com). Additionally, BLAST analysis using the probe sequence identified homologous transcript assemblies (TAs) within the TIGR website (http://blast.jcvi.org/euk-blast/plantta_blast.cgi) (specified for the Liliopsida). Genomic sequences were identified based on BLAST analysis against the high throughput genomic sequences (HTGS) and the genomic survey sequences (GSS). The open reading frames were identified (http://www.ncbi.nlm.nih.gov/projects/gorf/orfig.cgi) and thus the upstream 5’-sequences were deduced. Upstream regions (average length of 1kb) were scanned for cis-acting elements which were associated with hormone induction, modulation or responsiveness using the Plant-Cis-Acting-Elements (PLACE) software (http://www.dna.affrc.go.jp/PLACE/signalscan.html) [54,55].

### Fusarium head blight experiments

The two-row spring barley (*Hordeum vulgare* L.) cultivar (cv.) Lux was used in this study (kindly supplied by Powerseeds, Kildare, Ireland). This cultivar is susceptible to FHB [15]. All head blight experiments were conducted in a non-climate controlled glasshouse and plants were grown to mid-anthesis at which point heads were treated. In all experiments heads were covered with a polythene bag immediately after fungal/Tween20 treatment and plants were arranged in a randomised block design.

For experiments that analysed the effect of *P. fluorescens* on the accumulation of ABA and IAA in *F. culmorum-*infected heads, heads (two per plant) were treated with either bacterium, sterile water (controls), and 24 h later with either Tween20 or *F. culmorum,* all as previously described [17]. Each treatment combination was applied to 10 plants (2 heads per plant) per harvest time point and this experiment was conducted twice between May and August 2008. For experiments that investigated the effect of hormones on gene expression, plants were grown as above and, at mid-anthesis, heads (two per plant) were sprayed to runoff with either indole acetic acid (IAA), absciscic acid (ABA), (Sigma, UK) in dimethyl sulfoxide (DMSO; 10 μgml^-1^) or DMSO (10 μgml^-1^) alone. Twenty-four hours post-hormone applications, the same heads were treated with either Tween20 or *F. culmorum,* all as previously described by [17]. Each treatment combination was applied to four plants (2 heads per plant) per harvest time point and this experiment was conducted twice between May and August 2009. Heads were harvested at either 4, 12, 24 or 48 h post-fungal application, freeze-dried and stored at -70°C. Freeze-dried plant material was ground to a fine powder (the two heads harvested per plant were bulked) using a mortar and pestle and liquid nitrogen prior to either RNA or hormone extraction.

For experiments that investigated the effect of hormones on disease development, cv. Lux plants were grown and treated with either ABA or IAA and either pathogen or Tween 20 as above. Each treatment combination was applied to sixteen plants (2 heads per plant) and this experiment was conducted twice between Dec and June 2012. Disease was scored as the percentage of spikelets per head showing premature bleaching at growth stage (GS) 80 (start of dough development), as previously described [15]. At GS 90, heads were harvested, freeze-dried, and the 1000 grain weight was determined on per head basis.

### *In vitro* plate assay

Potato dextrose agar (PDA) was amended with either IAA or ABA (Sigma, UK). Hormones were dissolved in DMSO and added at a final concentration of either 0, 0.1, 1, 5, 10, 25, 50, 100 μg ml-^1^ PDA, DMSO concentration in all plates was adjusted to 1% wv^-1^. Plates were inoculated with 5 mm diameter plug of *F. culmorun* strain FCF200, which was harvested from a seven-day-old PDA plate. Plates were incubated at 25°C. After 72 h, the diameter of the colony (in cm) was measured. The experiment was carried out twice, each containing three technical replicate per treatmeant.

### Hormone analyses

Hormone extracts were prepared from 250mg plant material using the protocol described by Dobrev *et al.,*[56,57]. Indole acetic acid (IAA) and abscisic acid (ABA) were quantified by enzyme-linked immunosorbent analysis (ELISA) using the Olchemim C1 kits (Olchemim, Olomouc, Czech Republic). The procedure was conducted according to the manufacturer instructions, except that the incubation time was increased to 60 min. Each ELISA analysis included IAA or ABA standards (From 3.9 to 0.061 pmol) (Olchemim, Olomouc, Czech Republic). Using the OD (405 nm) absorbance values, hormone concentrations were extrapolated from a standard curve that related the absorbance to concentration of either the IAA or ABA standard.

### RNA extraction and semi-quantitative real time PCR

RNA was extracted from 200mg of plant material according to a modified hot-phenol procedure [58]. RNA was DNase-treated and quality was checked as previously described [17]. Real-time RT-PCR analysis was used to analyse the temporal expression of a selected subset of transcripts from the microarray studies completed previously [17]. Genes analysed were: a putative non-specific lipid transfer protein (*nsLTP*), a tonoplast aquaporin (*TIP3:1*), a subtilisin-chymotrypsin inhibitor (*CI-1B*), a zinc methallothionin-like protein (*ZnMT*), a serpin Z4 (*Paz1*), a nine-cis-epoxycarotenoid dioxygenase (*NCED*) and a signalling cascade protein (*Pi2*). The housekeeping gene used for normalisation of RT-PCR data was α-tubulin (GenBank accession no. AJ132399.1); real-time RT-PCR analysis validated its constitutive expression (irrespective of treatment; results not shown). Real-time quantification of target transcripts and of the housekeeping gene was performed in separate reactions. Primers and PCR conditions were previously described [17]. The threshold cycle (C_T_) values obtained by real-time RT-PCR were used to calculate the accumulation of target (relative mRNA accumulation), relative to α-tubulin transcript, with the formula 2 ^-(CT target transcript – CT α-tubulin)^[59].

### Statistical analysis

Statistical analysis of the real-time RT-PCR and the hormone data was performed using Minitab 16 (Minitab Inc., State College, Pennsylvania, USA). Data were tested for normal distribution using the Kolmogorov-Smirnov normality test. Non-normally distributed data were analysed using the Kruskal-Wallis and Mann–Whitney Rank sum tests. Normally distributed data was analysed using a one-way analysis of variance (ANOVA) incorporating Tukey’s pairwise comparison (5% level of significance).

## Competing interests

The authors declare that they have no competing interests.

## Authors’ contributions

CP carried out the head blight experiment, collected and processed the samples including RNA extraction and RT-PCR and drafted the manuscript. KR completed the RT analyses and helped with the hormone extraction and the drafting of the manuscript. SSA carried out the head blight trials for disease assessment and the plate assays. MB contributed to the sample collection and the RNA extraction. FD conceived the study, helped with the design and experimentation, and with the drafting of manuscript. All authors read and approved the final manuscript.

## Supplementary Material

Additional file 1 **Table S1.** Identification of hormone-responsive *cis*-acting elements within the 5’-region of barley genes potentiated by *Pseudomonas fluorescens* (strain MKB 158) to respond to attack by *Fusarium culmorum* (strain FCF200). Click here for file

Additional file 2 **Figure S1.** Effect of hormones on the in vitro growth of *Fusarium culmorum* (strain FCF200) on potato dextrose agar. Click here for file
