# Supplementary material for: Auxin as a player in the biocontrol of Fusarium head blight disease of barley and its potential as a disease control agent
Source: BMC Plant Biol. 2012 Nov 22;12:224. doi: 10.1186/1471-2229-12-224 (PMC3556313; doi:10.1186/1471-2229-12-224)
Supplement: Additional file 1 — Table S1. Identification of hormone-responsive cis-acting elements within the 5’-region of barley genes potentiated by Pseudomonas fluorescens (strain MKB 158) to respond to attack by Fusarium culmorum (strain FCF200). [file 1471-2229-12-224-S1.doc]

Table S1: Identification of hormone-responsive *cis*-acting elements within the 5’-region of barley genes*^ab^*.

| **Probe number** | **Genbank Accession** | **5’-genomic region(bp)** | **Annotation(Species)** | ***Cis*-acting elements** *^c^* | | **Hormone responsiveness***^c^* |
| --- | --- | --- | --- | --- | --- | --- |
|  |  |  |  | **name** | **sequence***^c^* |  |
| Contig22016_at | AC239018.1 | 1026 | Putative serine/threonine kinase protein (Rice) | CAREOSREP1  DPBFCOREDCDC3  ELRECOREPCRP1  LTRECOREATCOR15  MYB1AT  MYBATRD22  MYCCONSENSUSAT  PYRIMIDINEBOXOSRAMY1A  WBOXATNPR1  WRKY71OS | CAACTC  ACACNNG  TTGACC  CCGAC  WAACCA  CTAACC  CANNTG  CCTTTT  TTGAC  TGAC | GA  ABA  SA  ABA  ABA  ABA  ABA  GA  SA  GA |
| Contig5704_at | AC239093.1 | 1159 | ESTERASE lipase Gibberellin receptor GID1L2 (Maize) | ABREOSRAB21  ARFAT  DPBFCOREDCDC3  DRE2COREZMRAB17  GCCCORE  LTRECOREATCOR15  MYB1AT  MYCCONSENSUSAT  PYRIMIDINEBOXOSRAMY1A  SURECOREATSULTR11  WRKY71OS | ACGTSSSC TGTCTC ACACNNG  ACCGAC  GCCGCC  CCGAC WAACCA  CANNTG  CCTTTT  GAGAC  TGAC | ABA  AUX/ARF  ABA  ABA  JA  ABA  ABA  ABA  GA  AUX/ARF  GA |
| HT03K14r_s_at | AC239049.1 | 1024 | Aquaporin TIP3-1 (Maize) | DRE2COREZMRAB17  DPBFCOREDCDC3  LTRECOREATCOR15  WRKY71OS | ACACNNG  ACCGAC  CCGAC  TGAC | ABA  ABA  ABA  GA |
| Contig20_at | AC239076.1 | 910 | Histone H4(Rice) | ACGTABREMOTIFA2OSEM  AUXRETGA2GMGH3  GCCCORE  WRKY71OS | ACGTGKC  TGACGTGG  GCCGCC  TGAC | ABA  ABA  JA  GA |
| Contig4271_at | CZ885133.1 | 1270 | Cytochrome P450 CYP71C36 (Maize) | ABREATCONSENSUS  ACGTABREMOTIFA2OSEM  ASF1MOTIFCAMV  GAREAT  MYBGAHV  MYCATRD22  MYCCONSENSUSAT  PYRIMIDINEBOXOSRAMY1A  TATCCACHVAL21  WRKY71OS | YACGTGGC  ACGTGKC  TGACG  TAACAAR  TAACAAA  CACATG  CANNTG  CCTTTT  TATCCAC  TGAC | ABA  ABA  AUX  GA  GA  ABA  ABA  GA  GA  GA |
| Contig21968_at | AC239029.1 | 1239 | Glutathione-S-transferase Cla47 (Rice) | DPBFCOREDCDC3  ERELEE4  GARE2OSREP1  GCCCORE  LTRECOREATCOR15  MYCCONSENSUSAT  SURECOREATSULTR11  WBOXATNPR1  WRKY71OS | ACACNNG  AWTTCAAA  TAACGTA  GCCGCC  CCGAC  CANNTG  GAGAC  TTGAC  TGAC | ABA  ET  GA  JA  ABA  ABA  AUX/ARF  SA  GA |
| Contig12042_at | AC239010.1 | 1673 | Brassinosteroid C-3 oxidase (Barley) | ARFAT  ASF1MOTIFCAMV  CACGCAATGMGH3  DPBFCOREDCDC3  DRE2COREZMRAB17  GCCCORE  LTRECOREATCOR15  MYB2CONSENSUSAT  MYCCONSENSUSAT  SURECOREATSULTR11  WRKY71OS | TGTCTC  TGACG  CACGCAATACACNNG  ACCGAC  GCCGCC  CCGAC  YAACKG  CANNTG  GAGAC  TGAC | ARF  SA  AUX  ABA  ABA  ET  ABA  ABA  ABA  AUX/ARF  GA |
| Contig11361_at |  | 1087 | Peroxidase 51 (Maize) | ABREOSRAB21  ACGTABREMOTIFA2OSEM  SF1MOTIFCAMV  AUXREPSIAA4  DPBFCOREDCDC3  DRECRTCOREAT  GCCCORE  GGTCCCATGMSAUR  LTRECOREATCOR15  MYCCONSENSUSAT  SURECOREATSULTR11  TATCCACHVAL21  TATCCAOSAMY  WBOXATNPR1  WRKY71OS | ACGTSSSC  ACGTGKC  TGACG  KGTCCCAT  ACACNNG  RCCGAC  GCCGCC  GGTCCCAT  CCGAC  CANNTG  GAGAC  TATCCAC  TATCCA  TTGAC  TGAC | ABA  ABA  AUX/SA  aux  aba  aba  JA  AUX  ABA  ABA  AUX/ARF  GA  ga  SA  GA |
| Contig9764_at | AC239029.1 | 1233 | Glutathione-S-transferase Cla47 (Wheat) | ASF1MOTIFCAMV  DPBFCOREDCDC3  ERELEE4  GARE2OSREP1  GCCCORE  LTRECOREATCOR15  MYCCONSENSUSAT  SURECOREATSULTR11  WRKY71OS | TGACG  ACACNNG  AWTTCAAA  TAACGTA  GCCGCC  CCGAC  CANNTG  GAGAC  TGAC | SA  ABA  ET  GA  JA  ABA  ABA  ARF  JA |
| Mla12DH_orf_3pri12_s_at | AC239069.1 | 1345 | MLA12 (Barley) | ARFAT  ASF1MOTIFCAMV  CATATGGMSAUR  ERELEE4  GARE1OSREP1  GT1CONSENSUS  LTRECOREATCOR15  MYB1AT  MYCCONSENSUSAT  SURECOREATSULTR11  WBOXATNPR1  WRKY71OS | TGTCTC  TGACG  CATATG  AWTTCAAA  TAACAGA  GRWAAW  CCGAC  WAACCA  CANNTG  GAGAC  TTGAC  TGAC | ARF  SA  AUX  ET  GA  SA  ABA  ABA  ABA  AUX/ARF  SA  GA |
| Contig2112_at | CZ885936.1 | 1555 | Root peroxidase (Wheat) | ASF1MOTIFCAMV  DRE2COREZMRAB17  LTRECOREATCOR15  MYB1AT  MYB2CONSENSUSAT  WRKY71OS | TGACG  ACCGAC  CCGAC  WAACCA  YAACKG  TGAC | SA  ABA  ABA  ABA  ABA  GA |
| Rbah13p07_s_at | CZ885761.1 | 710 | Root peroxidase (Wheat) | DRE2COREZMRAB17  LTRECOREATCOR15  MYB1AT  MYB2CONSENSUSAT  WRKY71OS | ACCGAC  CCGAC  WAACCA  YAACKG  TGAC | ABA  ABA  ABA  ABA  GA |
| Contig1877_at | AC239019.1 | 1450 | SerpinZ4 | ABREOSRAB21  ACGTABREMOTIFA2OSEM  DPBFCOREDCDC3  ELRECOREPCRP1  GAREAT  GCCCORE  MYB1AT  MYB2CONSENSUSAT  MYCCONSENSUSAT  SURECOREATSULTR11  WBOXATNPR1  WRKY71OS | ACGTSSSC  ACGTGKC  ACACNNG  TTGACC  TAACAAR  GCCGCC  WAACCA  YAACKG  CANNTG  GAGAC  TTGAC  TGAC | ABA  ABA  ABA  SA  GA  SA  ABA  ABA  ABA  AUX/ARF  SA  GA |
| Contig1868_s_at | AC239038.1 | 1095 | Peroxidase BP1 | ABREOSRAB21  ABRERATCAL  ABREZMRAB28  ACGTABREMOTIFA2OSEM  AGCBOXNPGLB  ASF1MOTIFCAMV  AUXREPSIAA4  DPBFCOREDCDC3  DRE2COREZMRAB17  GCCCORE  GGTCCCATGMSAUR  LTRECOREATCOR15  MYB1AT  MYBATRD22  MYCATRD22  MYCCONSENSUSAT  PYRIMIDINEBOXHVEPB1  SURECOREATSULTR11  TATCCACHVAL21  TATCCAOSAMY TATCCA  WBOXATNPR1  WRKY71OS | ACGTSSSC  MACGYGB  CCACGTGG  ACGTGKC  AGCCGCC  TGACG  KGTCCCAT  ACACNNG  ACCGAC  GCCGCC  GGTCCCAT  CCGAC  WAACCA  CTAACCA  CACATG  CANNTG  TTTTTTCC  GAGAC  TATCCAC  TATCCA  TTGAC  TGAC | ABA  ABA  ABA  ABA  ET  AUX  Aux, SA  ABA  ABA  JA  AUX  ABA  ABA  ABA  ABA  ABA  GA, ABA  AUX/ARF  GA  GA  SA  GA |
| Contig960_s_at | AC239045.1 | 870 | Chlorophyll a-b binding protein 3A (Tomato) | LTRECOREATCOR15  WRKY71OS | CCGAC  TGAC | ABA  GA |
| Contig13304_at | AC239076.1 | 976 | Desiccation-related protein PCC13-62 (Maize) | AGCBOXNPGLB  ASF1MOTIFCAMV  DPBFCOREDCDC3  DRE1COREZMRAB17  GCCCORE  MYCCONSENSUSAT  SURECOREATSULTR11  WBOXHVISO1  WRKY71OS | AGCCGCC  TGACG  ACACNNG  ACCGAGA  GCCGCC  CANNTG  GAGAC  TGACT  TGAC | ERF  SA  ABA  ABA  JA  ABA  AUX/ARF  SA  JA |
| HV_CEa0001E19r2_at | AK357937.1 | 790 | Putative lysine decarboxylase (Banana) | AGCBOXNPGLB  GCCCORE  WBOXATNPR1  WRKY71OS | AGCCGCC  GCCGCC  TTGAC  TGAC | ET  JA  SA  GA |
| Contig6910_at | CZ885357.1 | 766 | Patatin-like protein 1 (Tobacco) | ASF1MOTIFCAMV  RYREPEATVFLEB4  WBOXATNPR1  WRKY71OS | TGACG  CATGCATG  TTGAC  TGAC | SA  ABA  SA  GA |
| HZ01L11u_at | AC239088.1 | 677 | Glycosyltransferase family 28 N-terminal domain containing protein (Rice) | DPBFCOREDCDC3  ELRECOREPCRP1  LTRECOREATCOR15  YCATRD22  MYCCONSENSUSAT  SBOXATRBCS  SURECOREATSULTR11  WBOXATNPR1  WRKY71OS | ACACNNG  TTGACC  CCGAC  CACATG  CANNTG  CACCTCCA  GAGAC  TTGAC  TGAC | ABA  SA  ABA  ABA  ABA  ABA  AUX/ARF  SA  GA |
| Contig23510_at | AC239087.1 | 1371 | Hydrolase, hydrolyzing O-glycosyl compounds (Maize) | ASF1MOTIFCAMV  ELRECOREPCRP1  GAREAT  GCCCORE  MYB1AT  MYBGAHV  MYCCONSENSUSAT  WBOXATNPR1  WRKY71OS | TGACG  TTGACC  TAACAAR  GCCGCC  WAACCA  TAACAAA  CANNTG  TTGAC  TGAC | SA  SA  GA  JA  ABA  GA  ABA  SA  GA |
| Contig20187_s_at | AC239024.1 | 1233 | Serine carboxypeptidase 1 (Maize) | ARFAT  GARE2OSREP1  LTRECOREATCOR15  MYB1AT  MYB2CONSENSUSAT  SURECOREATSULTR11  WRKY71OS | TGTCTC  TAACGTA  CCGAC  WAACCA  YAACKG  GAGAC  TGAC | AUX  GA  ABA  ABA  ABA  AUX/ARF  GA |
| Contig13973_at | AC239070.1 | 1437 | CBL-interacting serine/threonine-protein kinase 15 (Maize) | CATATGGMSAUR  DPBFCOREDCDC3  DRE2COREZMRAB17  ERELEE4  LTRECOREATCOR15  MYB1AT  MYCCONSENSUSAT  SURECOREATSULTR11  WRKY71OS | CATATG  ACACNNG  ACCGAC  AWTTCAAA  CCGAC  WAACCA  CANNTG  GAGAC  TGAC | AUX  ABA  ABA  ET  ABA  ABA  ABA  AUX/ARF  GA |
| Contig15583_at | AC239077.1 | 1109 | Avenin-like protein (Wheat) | CATATGGMSAUR  DPBFCOREDCDC3  DRE2COREZMRAB17  LTRECOREATCOR15  MYCCONSENSUSAT  NTBBF1ARROLB  PYRIMIDINEBOXOSRAMY1A  WRKY71OS | CATATG  ACACNNG  ACCGAC  CCGAC  CANNTG  ACTTTA  CCTTTT  TGAC | AUX  ABA  ABA  ABA  ABA  AUX  GA  GA |
| Contig8710_at | AC239006.1 | 1767 | Globulin (Barley) | ARFAT  ASF1MOTIFCAMV  DPBFCOREDCDC3  GCCCORE  LTRECOREATCOR15  MYBST1  MYCCONSENSUSAT  NTBBF1ARROLB  PYRIMIDINEBOXOSRAMY1A  GASURECOREATSULTR11  TCA1MOTIF  WRKY71OS | TGTCTC  TGACG  ACACNNG  GCCGCC  CCGAC  GGATA  CANNTG  ACTTTA  CCTTTT  GAGAC  TCATCTTCT  TGAC | ARF  AUX, SA  ABA  JA  ABA  ABA  ABA  AUX  GA  AUX/ARF  SA  GA |
| Contig241_x_at | AC239077.1 | 1700 | B1 Hordein (Barley) | CATATGGMSAUR  DPBFCOREDCDC  DRE2COREZMRAB17  LTRECOREATCOR15  MYCATRD22  MYCCONSENSUSAT  PYRIMIDINEBOXOSRAMY1A  TATCCACHVAL21  TATCCAOSAMY  WRKY71OS | CATATG  ACACNNG  ACCGAC  CCGAC  CACATG  CANNTG  CCTTTT  TATCCAC  TATCCA  TGAC | AUX/ARF  ABA  ABA  ABA  ABA  ABA  GA  GA  GA  GA |
| Contig585_x_at | AC239077.1 | 1000 | B1 Hordein (Barley) | CATATGGMSAUR  DPBFCOREDCDC3  DRE2COREZMRAB17  LTRECOREATCOR15  MYCATRD22  MYCCONSENSUSAT  PYRIMIDINEBOXOSRAMY1A  WRKY71OS | CATATG  ACACNNG  ACCGAC  CCGAC  CACATG  CANNTG  CCTTTT  TGAC | AUX  ABA  ABA  ABA  ABA  ABA  GA  GA |
| Contig282_x_at | AC239077.1 | 1000 | B1 hordein (Barley) | ARFAT  DPBFCOREDCDC3  ASF1MOTIFCAMV  LTRECOREATCOR15  MYB1AT  MYB2CONSENSUSAT  MYCCONSENSUSAT  PROXBBNNAPA  SURECOREATSULTR11  WRKY71OS | TGTCTC  TGACG  ACACNNG  CCGAC  WAACCA  YAACKG  CANNTG  CAAACACC  GAGAC  TGAC | ARF  SA  ABA  ABA  ABA  ABA  ABA  ABA  AUX/ARF  GA |
| Contig530_x_at | AC239077.1 | 1000 | B hordein (Barley) | CATATGGMSAUR  DPBFCOREDCDC3  DRE2COREZMRAB17  LTRECOREATCOR15  MYCCONSENSUSAT  PYRIMIDINEBOXOSRAMY1A  WRKY71OS | CATATG  ACACNNG  ACCGAC  CCGAC  CANNTG  CCTTTT  TGAC | AUX  ABA  ABA  ABA  ABA  GA  GA |
| Contig524_x_at | AC239077.1 | 1000 | B1 hordein (Barley) | CATATGGMSAUR  DPBFCOREDCDC3  MYCCONSENSUSAT  PYRIMIDINEBOXOSRAMY1A | CATATG  ACACNNG  CANNTG  CCTTTT | AUX  ABA  ABA  GA |
| Contig502_x_at | AC239077.1 | 1000 | B hordein (Barley) | CATATGGMSAUR  DPBFCOREDCDC3  MYCCONSENSUSAT  PYRIMIDINEBOXOSRAMY1A | CATATG  ACACNNG  CANNTG  CCTTTT | AUX  ABA  ABA  GA |
| HVSMEl0020J06r2_at | CZ885501.1 | 700 | PDR-type ABC transporter (Wheat) | ASF1MOTIFCAMV  LTRECOREATCOR15  MYCATRD22  MYCCONSENSUSAT  WRKY71OS | TGACG  CCGAC  CACATG  CANNTG  TGAC | SA  ABA  ABA  ABA  GA |
| Contig19947_s_at | EU176161.1 | 1233 | AMP-binding protein (Maize) | AGCBOXNPGLB  ASF1MOTIFCAMV  DPBFCOREDCDC3  GCCCORE  LTRECOREATCOR15  MYB2CONSENSUSAT  MYCATRD22  MYCCONSENSUSAT  T/GBOXATPIN2  WBOXATNPR1  WRKY71OS | AGCCGCC  TGACG  ACACNNG  GCCGCC  CCGAC  YAACKG  CACATG  CANNTG  AACGTG  TTGAC  TGAC | ET  SA  ABA  JA  ABA  ABA  ABA  ABA  JA  SA  GA |
| Contig18112_at | AC239015.1 | 770 | Putative uncharacterized protein (Vitis) | ARFAT  DPBFCOREDCDC3  GCCCORE  LTRECOREATCOR15  MYB2CONSENSUSAT  SURECOREATSULTR11  WRKY71OS | TGTCTC  ACACNNG  GCCGCC  CCGAC  YAACKG  GAGAC  TGAC | ARF  ABA  JA  ABA  ABA  AUX/ARF  GA |
| Contig12203_at | CZ885425.1 | 535 | Vegetative cell wall protein gp1 (Maize) | MYB1AT  MYCCONSENSUSAT  PYRIMIDINEBOXOSRAMY1A  SURECOREATSULTR11  WRKY71OS | WAACCA  CANNTG  CCTTTT  GAGAC  TGAC | ABA  ABA  GA  AUX/ARF  GA |
| Contig245_s_at | AC239077.1 | 1100 | Putative uncharacterized protein (Psathyrostachys huashanica) | MYB1AT  MYCCONSENSUSAT  WRKY71OS | WAACCA  CANNTG  TGAC | ABA  ABA  GA |
| Rbaal19a12_at | AC239045.1 | 1233 | Uncharacterized (Rice) | ARFAT  ELRECOREPCRP1  MYB1AT  MYB2CONSENSUSAT  MYCCONSENSUSAT  SURECOREATSULTR11  TATCCAOSAMY  WBOXATNPR1  WRKY71OS | TGTCTC  TTGACC  WAACCA  YAACKG  CANNTG  GAGAC  TATCCA  TTGAC  TGAC | ARF  SA  ABA  ABA  ABA  AUX/ARF  GA  SA  GA |
| Contig17275_at | AF521177.1 | 1100 | No hits | DPBFCOREDCDC3  GCCCORE  LTRECOREATCOR15  MYCCONSENSUSAT  SURECOREATSULTR11  WRKY71OS | ACACNNG  GCCGCC  CCGAC  CANNTG  GAGAC  TGAC | ABA  JA  ABA  ABA  AUX/ARF  GA |
| HI07N13r_at | AC239092.1 | 780 | No hits | CATATGGMSAUR  MYB1AT  MYB2CONSENSUSAT  MYCCONSENSUSAT  WBOXATNPR1  WRKY71OS | CATATG  WAACCA  YAACKG  CANNTG  TTGAC  TGAC | AUX  ABA  ABA  ABA  SA  GA |
| Contig20334_at | AC239071.1 | 870 | No hits | GCCCORE  MYCCONSENSUSAT  SURECOREATSULTR11  WBOXATNPR1  WRKY71OS | GCCGCC  CANNTG  GAGAC  TTGAC  TGAC | JA  ABA  AUX/ARF  SA  GA |

*^a^*Potentiated by *Pseudomonas fluorescens* to transcriptionally respond to the pathogen *Fusarium culmorum.*

*^b^*Only potentiated genes whereby genomics equivalent were identified where enclosed in the table.

*^c^*Cis-acting elements, motifs and hormone responsiveness where identified through the PLACE database.
