# Supplementary material for: Auxin as a player in the biocontrol of Fusarium head blight disease of barley and its potential as a disease control agent
Source: BMC Plant Biol. 2012 Nov 22;12:224. doi: 10.1186/1471-2229-12-224 (PMC3556313; doi:10.1186/1471-2229-12-224)
Supplement: Additional file 2 — Figure S1. Effect of hormones on the in vitro growth of Fusarium culmorum (strain FCF200) on potato dextrose agar. [file 1471-2229-12-224-S2.docx]

**Additional figure S1**


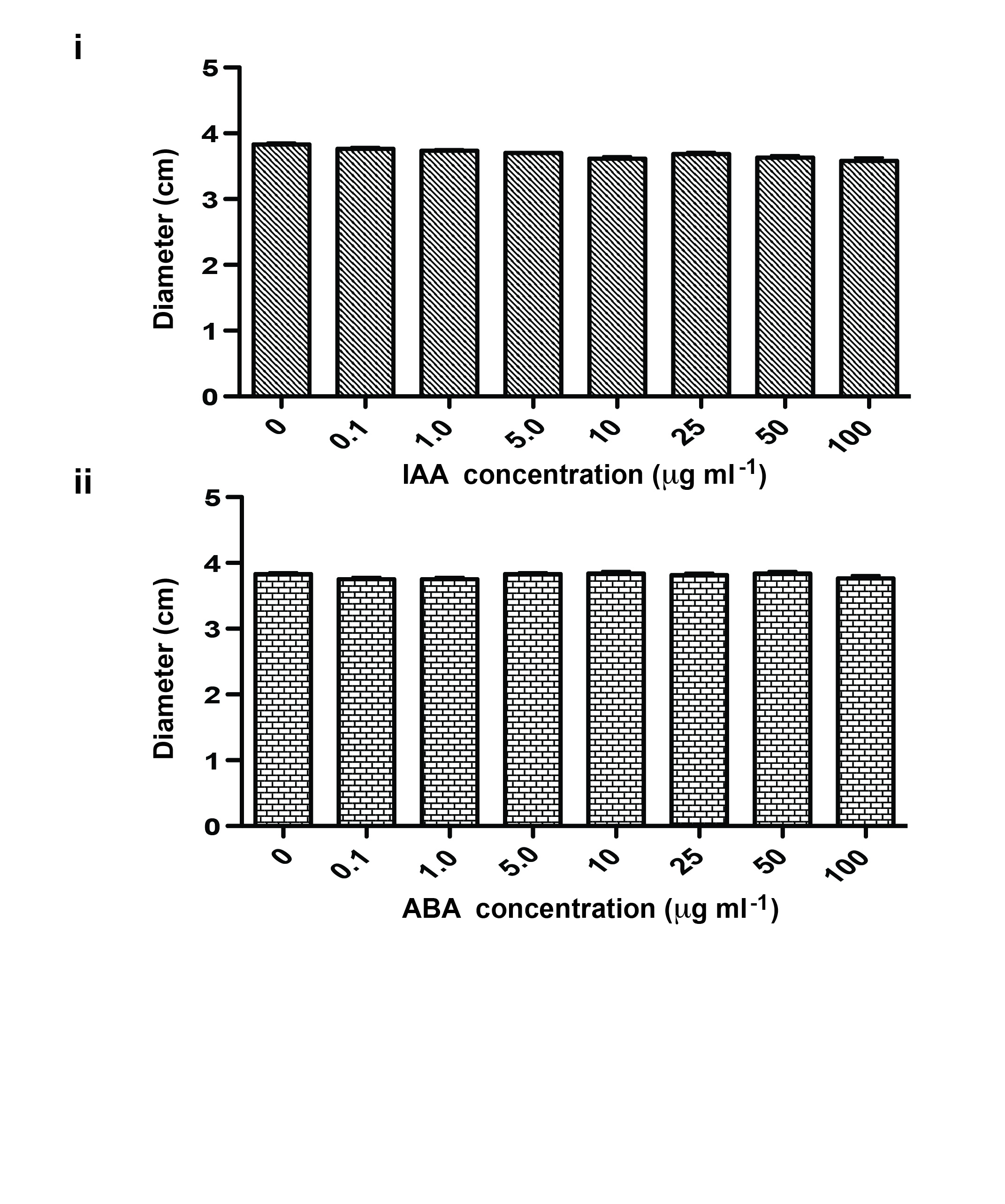


**Fig. S1.** Effect of hormones on the *in vitro* growth of *Fusarium culmorum* (strain FCF200) on potato dextrose agar. Plates were amended with either indole acetic acid (IAA) or abscisic acid (ABA) (from 0 to 100 ug ml^-1^). After 72h incubation at at 25° C, colony diameter was measured (in cm). Results are based on two experiments, each including three plate per treatment and bars indicate the standard error of the mean.
